# *N*-Cyclo­hexyl-4-[(2-nitro­anilino)­meth­yl]thio­phene-2-sulfonamide

**DOI:** 10.1107/S160053681103861X

**Published:** 2011-09-30

**Authors:** Yu-xin He, Rong-sheng Tong, Jin-wei Wu, Zou Jing, Jian-you Shi

**Affiliations:** a Bioengineering College, Xihua University, Chengdu, Sichuan 610039, People’s Republic of China; b Sichuan Academy of Medical Sciences and, Sichuan Provincial People’s Hospital, Chengdu, Sichuan 610072, People’s Republic of China

## Abstract

In the title compound, C_17_H_21_N_3_O_4_S_2_, an intra­molecular N—H⋯O hydrogen bond involving the proximate amine and nitro groups is observed. In the crystal, inter­molecular N—H⋯O hydrogen bonds involving the amine and SO_2_ groups occur. One of the notro O atoms is disordered over two conformations with occupancies of 0.578 (12) and 0.422 (12).

## Related literature

For uses of thio­phene-2-sulfonamides, see: Cuberes Altisen *et al.* (2007 ▶); Santhakumar & Tomaszewski (2006 ▶).
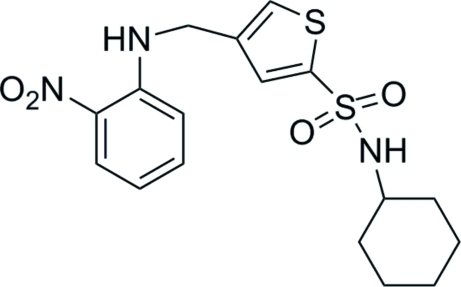


## Experimental

### 

#### Crystal data

C_17_H_21_N_3_O_4_S_2_
*M* *_r_* = 395.49
Monoclinic, 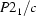

*a* = 7.4201 (2) Å
*b* = 27.8329 (7) Å
*c* = 10.1790 (3) Å
β = 118.440 (3)°
*V* = 1848.50 (10) Å^3^
*Z* = 4
Mo *K*α radiation
μ = 0.32 mm^−1^
*T* = 145 K
0.38 × 0.25 × 0.15 mm

#### Data collection

Agilent Xcalibur Eos diffractometer
Absorption correction: multi-scan (*CrysAlis PRO*; Agilent, 2011 ▶) *T*_min_ = 0.970, *T*_max_ = 1.0
8048 measured reflections
3772 independent reflections
3104 reflections with *I* > 2σ(*I*)
*R*_int_ = 0.021

#### Refinement

*R*[*F* ^2^ > 2σ(*F* ^2^)] = 0.058
*wR*(*F* ^2^) = 0.129
*S* = 1.01
3772 reflections
225 parameters
1 restraint
H atoms treated by a mixture of independent and constrained refinement
Δρ_max_ = 1.02 e Å^−3^
Δρ_min_ = −0.68 e Å^−3^

### 

Data collection: *CrysAlis PRO* (Agilent, 2011 ▶); cell refinement: *CrysAlis PRO*; data reduction: *CrysAlis PRO*; program(s) used to solve structure: *SHELXS97* (Sheldrick, 2008 ▶); program(s) used to refine structure: *SHELXL97* (Sheldrick, 2008 ▶); molecular graphics: *OLEX2* (Dolomanov *et al.*, 2009 ▶); software used to prepare material for publication: *OLEX2*.

## Supplementary Material

Crystal structure: contains datablock(s) global, I. DOI: 10.1107/S160053681103861X/bv2190sup1.cif

Structure factors: contains datablock(s) I. DOI: 10.1107/S160053681103861X/bv2190Isup2.hkl

Supplementary material file. DOI: 10.1107/S160053681103861X/bv2190Isup3.cml

Additional supplementary materials:  crystallographic information; 3D view; checkCIF report

## Figures and Tables

**Table 1:** Hydrogen-bond geometry (Å, °)

*D*—H⋯*A*	*D*—H	H⋯*A*	*D*⋯*A*	*D*—H⋯*A*
N1—H1⋯O1^i^	0.88	2.27	2.956 (3)	134
N2—H2⋯O2^ii^	0.81 (3)	2.36 (3)	2.979 (3)	135 (3)
N2—H2⋯O3	0.81 (3)	2.01 (3)	2.620 (4)	132 (3)

Symmetry codes: (i) 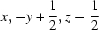; (ii) 
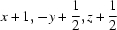
.

**Fig. 1.**
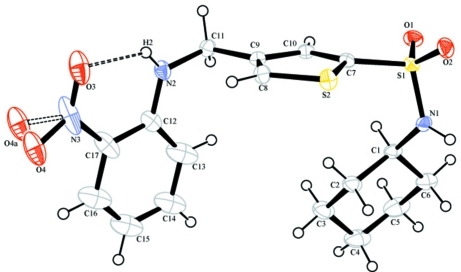
The molecular structure of title compound. The intramolecular N-H···O hydrogen bond is shown by dashed lines.

## supplementary crystallographic information

### Comment

*N*-Cyclohexyl-4-((2-nitrophenylamino)methyl)thiophene-2-sulfonamide is potentially useful in the treatment of a condition mediated by the CB2 receptor (Cuberes Altisen, *et al.*, 2007). In the title compound, C_17_H_21_N_3_O_4_S_2_, there is an intramolecular N-H···O between the amine and nitro groups as well as N-H···O intermolecular hydrogen bonds involving the amine and SO_2_ moieties. O4 is disordered over two conformations with occupancies of 0.578 (12) and 0.422 (12).

### Experimental

A mixture of 4-((2-nitrophenylamino)methyl)thiophene-2-sulfonyl chloride (3.22 g, 0.01 mol), trimethylamine (0.885 g, 0.015 mol) and cyclohexanamine (0.99 g, 0.01 mol) in THF (150 mL) was heated to reflux for 3 h, then the solvent removed and the residue separated by silica gel column to obtain the target compound. Single crystals were obtained from the powder in CH_2_Cl_2_ and methanol after 3 days.

### Refinement

H atoms were placed in geometrically idealized positions and constrained to ride on their parent atoms with a C—H distances of 0.95 to 1.00 Å [*U*_iso_(H) = 1.2*U*_eq_(C)].  The H atoms attached to N were idealized with N–H distances of 0.88 Å. H2 atom located by difference fouraer map.

### Crystal data

**Table:**

C_17_H_21_N_3_O_4_S_2_	*F*(000) = 832
*M**_r_* = 395.49	*D*_x_ = 1.421 Mg m^−^^3^
Monoclinic, *P*2_1_/*c*	Mo *K*α radiation, λ = 0.7107 Å
Hall symbol: -P 2ybc	Cell parameters from 3479 reflections
*a* = 7.4201 (2) Å	θ = 2.9–29.1°
*b* = 27.8329 (7) Å	µ = 0.32 mm^−^^1^
*c* = 10.1790 (3) Å	*T* = 145 K
β = 118.440 (3)°	Block, yellow
*V* = 1848.50 (10) Å^3^	0.38 × 0.25 × 0.15 mm
*Z* = 4	

### Data collection

**Table:**

Agilent Xcalibur Eos diffractometer	3772 independent reflections
Radiation source: fine-focus sealed tube	3104 reflections with *I* > 2σ(*I*)
graphite	*R*_int_ = 0.021
Detector resolution: 16.0874 pixels mm^-1^	θ_max_ = 26.4°, θ_min_ = 2.9°
ω scans	*h* = −9→9
Absorption correction: multi-scan (*CrysAlis PRO*; Agilent, 2011)	*k* = −31→34
*T*_min_ = 0.970, *T*_max_ = 1.0	*l* = −12→10
8048 measured reflections	

### Refinement

**Table:**

Refinement on *F*^2^	Primary atom site location: structure-invariant direct methods
Least-squares matrix: full	Secondary atom site location: difference Fourier map
*R*[*F*^2^ > 2σ(*F*^2^)] = 0.058	Hydrogen site location: inferred from neighbouring sites
*wR*(*F*^2^) = 0.129	H atoms treated by a mixture of independent and constrained refinement
*S* = 1.01	*w* = 1/[σ^2^(*F*_o_^2^) + (0.0385*P*)^2^ + 3.9868*P*] where *P* = (*F*_o_^2^ + 2*F*_c_^2^)/3
3772 reflections	(Δ/σ)_max_ < 0.001
225 parameters	Δρ_max_ = 1.02 e Å^−^^3^
1 restraint	Δρ_min_ = −0.68 e Å^−^^3^

### Special details

**Table:**

Geometry. All e.s.d.'s (except the e.s.d. in the dihedral angle between two l.s. planes) are estimated using the full covariance matrix. The cell e.s.d.'s are taken into account individually in the estimation of e.s.d.'s in distances, angles and torsion angles; correlations between e.s.d.'s in cell parameters are only used when they are defined by crystal symmetry. An approximate (isotropic) treatment of cell e.s.d.'s is used for estimating e.s.d.'s involving l.s. planes.
Refinement. Refinement of *F*^2^ against ALL reflections. The weighted *R*-factor *wR* and goodness of fit *S* are based on *F*^2^, conventional *R*-factors *R* are based on *F*, with *F* set to zero for negative *F*^2^. The threshold expression of *F*^2^ > σ(*F*^2^) is used only for calculating *R*-factors(gt) *etc*. and is not relevant to the choice of reflections for refinement. *R*-factors based on *F*^2^ are statistically about twice as large as those based on *F*, and *R*- factors based on ALL data will be even larger.

### Fractional atomic coordinates and isotropic or equivalent isotropic displacement parameters (Å^2^)

**Table:**

	*x*	*y*	*z*	*U*_iso_*/*U*_eq_	Occ. (<1)
S1	−0.35683 (10)	0.25100 (2)	0.68116 (8)	0.02193 (18)	
S2	0.04835 (11)	0.23017 (3)	0.69685 (8)	0.02609 (19)	
O1	−0.4402 (3)	0.24711 (8)	0.7824 (2)	0.0303 (5)	
O2	−0.3343 (3)	0.29724 (7)	0.6291 (2)	0.0321 (5)	
O3	0.8016 (4)	0.10467 (11)	1.0716 (4)	0.0578 (8)	
O4	0.8590 (14)	0.0450 (3)	0.9436 (13)	0.060 (2)	0.578 (12)
O4A	0.9294 (19)	0.0474 (5)	1.0238 (16)	0.060 (2)	0.422 (12)
N1	−0.4979 (4)	0.21961 (8)	0.5357 (3)	0.0237 (5)	
H1	−0.5543	0.2332	0.4469	0.028*	
N2	0.4249 (4)	0.11205 (9)	1.0276 (3)	0.0284 (6)	
H2	0.538 (5)	0.1238 (12)	1.068 (4)	0.029 (9)*	
N3	0.7563 (6)	0.06859 (12)	0.9914 (5)	0.0608 (11)	
C1	−0.5341 (5)	0.16781 (10)	0.5495 (3)	0.0259 (6)	
H1A	−0.5033	0.1617	0.6550	0.031*	
C2	−0.3928 (5)	0.13607 (11)	0.5162 (3)	0.0331 (7)	
H2B	−0.2481	0.1438	0.5867	0.040*	
H2A	−0.4164	0.1425	0.4135	0.040*	
C3	−0.4336 (6)	0.08302 (12)	0.5313 (4)	0.0461 (9)	
H3A	−0.3467	0.0629	0.5038	0.055*	
H3B	−0.3961	0.0760	0.6366	0.055*	
C4	−0.6567 (7)	0.07034 (13)	0.4319 (4)	0.0552 (11)	
H4B	−0.6799	0.0364	0.4491	0.066*	
H4A	−0.6898	0.0737	0.3259	0.066*	
C5	−0.7976 (6)	0.10240 (14)	0.4624 (4)	0.0510 (10)	
H5B	−0.7766	0.0960	0.5645	0.061*	
H5A	−0.9419	0.0947	0.3908	0.061*	
C6	−0.7576 (5)	0.15573 (12)	0.4483 (4)	0.0348 (7)	
H6A	−0.7938	0.1630	0.3434	0.042*	
H6B	−0.8449	0.1757	0.4757	0.042*	
C7	−0.1161 (4)	0.22402 (10)	0.7707 (3)	0.0204 (6)	
C8	0.2109 (4)	0.18895 (11)	0.8212 (3)	0.0249 (6)	
H8	0.3358	0.1792	0.8246	0.030*	
C9	0.1434 (4)	0.17152 (10)	0.9146 (3)	0.0212 (6)	
C10	−0.0482 (4)	0.19163 (10)	0.8847 (3)	0.0210 (6)	
H10	−0.1204	0.1835	0.9376	0.025*	
C11	0.2639 (4)	0.13708 (11)	1.0413 (3)	0.0256 (6)	
H11B	0.3255	0.1552	1.1365	0.031*	
H11A	0.1685	0.1131	1.0459	0.031*	
C12	0.3906 (5)	0.07546 (11)	0.9310 (4)	0.0316 (7)	
C13	0.1906 (8)	0.05721 (14)	0.8436 (5)	0.0589 (6)	
H13	0.0794	0.0724	0.8485	0.071*	
C14	0.1522 (8)	0.01803 (13)	0.7514 (5)	0.0589 (6)	
H14	0.0160	0.0064	0.6951	0.071*	
C15	0.3109 (8)	−0.00463 (14)	0.7402 (5)	0.0589 (6)	
H15	0.2833	−0.0319	0.6774	0.071*	
C16	0.5047 (8)	0.01209 (13)	0.8186 (5)	0.0589 (6)	
H16	0.6129	−0.0034	0.8103	0.071*	
C17	0.5473 (6)	0.05249 (12)	0.9128 (5)	0.0450 (9)	

### Atomic displacement parameters (Å^2^)

**Table:**

	*U*^11^	*U*^22^	*U*^33^	*U*^12^	*U*^13^	*U*^23^
S1	0.0235 (3)	0.0198 (3)	0.0232 (4)	0.0021 (3)	0.0117 (3)	−0.0014 (3)
S2	0.0281 (4)	0.0275 (4)	0.0287 (4)	0.0020 (3)	0.0183 (3)	0.0070 (3)
O1	0.0291 (11)	0.0366 (12)	0.0302 (11)	0.0012 (9)	0.0182 (9)	−0.0082 (9)
O2	0.0316 (12)	0.0192 (10)	0.0380 (12)	0.0023 (9)	0.0106 (10)	0.0031 (9)
O3	0.0391 (15)	0.0464 (17)	0.096 (2)	0.0100 (13)	0.0383 (16)	0.0169 (16)
O4	0.050 (5)	0.057 (2)	0.095 (6)	0.017 (3)	0.052 (5)	0.011 (5)
O4A	0.050 (5)	0.057 (2)	0.095 (6)	0.017 (3)	0.052 (5)	0.011 (5)
N1	0.0280 (12)	0.0215 (12)	0.0181 (12)	0.0003 (10)	0.0082 (10)	0.0022 (9)
N2	0.0227 (13)	0.0229 (13)	0.0393 (15)	0.0004 (11)	0.0145 (12)	0.0007 (11)
N3	0.065 (2)	0.0361 (19)	0.119 (3)	0.0221 (17)	0.075 (2)	0.036 (2)
C1	0.0364 (16)	0.0231 (15)	0.0187 (14)	−0.0042 (12)	0.0135 (13)	0.0003 (11)
C2	0.0482 (19)	0.0268 (16)	0.0243 (15)	0.0024 (14)	0.0171 (15)	0.0002 (13)
C3	0.073 (3)	0.0254 (18)	0.0374 (19)	0.0041 (17)	0.0239 (19)	−0.0005 (15)
C4	0.092 (3)	0.0248 (18)	0.039 (2)	−0.017 (2)	0.024 (2)	−0.0056 (15)
C5	0.065 (3)	0.043 (2)	0.039 (2)	−0.027 (2)	0.0201 (19)	−0.0041 (17)
C6	0.0415 (18)	0.0339 (18)	0.0296 (17)	−0.0096 (15)	0.0176 (15)	−0.0042 (14)
C7	0.0225 (13)	0.0200 (14)	0.0219 (14)	−0.0017 (11)	0.0132 (11)	−0.0028 (11)
C8	0.0219 (14)	0.0269 (15)	0.0289 (15)	0.0006 (12)	0.0144 (12)	0.0016 (12)
C9	0.0224 (13)	0.0218 (14)	0.0197 (13)	−0.0038 (11)	0.0104 (11)	−0.0029 (11)
C10	0.0234 (14)	0.0227 (14)	0.0193 (13)	−0.0034 (11)	0.0122 (11)	−0.0018 (11)
C11	0.0239 (14)	0.0281 (16)	0.0248 (15)	0.0008 (12)	0.0117 (12)	0.0022 (12)
C12	0.0455 (18)	0.0206 (15)	0.0385 (18)	−0.0004 (14)	0.0281 (15)	0.0079 (13)
C13	0.1099 (19)	0.0302 (10)	0.0576 (13)	−0.0114 (11)	0.0569 (14)	−0.0012 (9)
C14	0.1099 (19)	0.0302 (10)	0.0576 (13)	−0.0114 (11)	0.0569 (14)	−0.0012 (9)
C15	0.1099 (19)	0.0302 (10)	0.0576 (13)	−0.0114 (11)	0.0569 (14)	−0.0012 (9)
C16	0.1099 (19)	0.0302 (10)	0.0576 (13)	−0.0114 (11)	0.0569 (14)	−0.0012 (9)
C17	0.075 (3)	0.0216 (16)	0.068 (3)	0.0064 (17)	0.058 (2)	0.0131 (16)

### Geometric parameters (Å, °)

**Table:**

S1—O1	1.436 (2)	C4—H4A	0.9900
S1—O2	1.431 (2)	C4—C5	1.515 (6)
S1—N1	1.605 (2)	C5—H5B	0.9900
S1—C7	1.742 (3)	C5—H5A	0.9900
S2—C7	1.720 (3)	C5—C6	1.534 (5)
S2—C8	1.708 (3)	C6—H6A	0.9900
O3—N3	1.235 (5)	C6—H6B	0.9900
O4—N3	1.265 (9)	C7—C10	1.362 (4)
O4A—N3	1.305 (12)	C8—H8	0.9500
N1—H1	0.8800	C8—C9	1.358 (4)
N1—C1	1.485 (4)	C9—C10	1.420 (4)
N2—H2	0.81 (3)	C9—C11	1.511 (4)
N2—C11	1.446 (4)	C10—H10	0.9500
N2—C12	1.353 (4)	C11—H11B	0.9900
N3—C17	1.437 (6)	C11—H11A	0.9900
C1—H1A	1.0000	C12—C13	1.412 (6)
C1—C2	1.528 (4)	C12—C17	1.414 (5)
C1—C6	1.515 (4)	C13—H13	0.9500
C2—H2B	0.9900	C13—C14	1.377 (5)
C2—H2A	0.9900	C14—H14	0.9500
C2—C3	1.530 (4)	C14—C15	1.387 (6)
C3—H3A	0.9900	C15—H15	0.9500
C3—H3B	0.9900	C15—C16	1.353 (6)
C3—C4	1.514 (6)	C16—H16	0.9500
C4—H4B	0.9900	C16—C17	1.412 (5)
			
S1—N1—H1	119.7	C4—C5—C6	111.5 (3)
S2—C7—S1	119.50 (16)	H4B—C4—H4A	108.0
S2—C8—H8	123.4	C5—C4—H4B	109.3
O1—S1—N1	107.83 (13)	C5—C4—H4A	109.3
O1—S1—C7	106.07 (13)	C5—C6—H6A	109.6
O2—S1—O1	119.95 (13)	C5—C6—H6B	109.6
O2—S1—N1	106.73 (13)	H5B—C5—H5A	108.0
O2—S1—C7	108.05 (13)	C6—C1—H1A	108.0
O3—N3—O4	130.6 (6)	C6—C1—C2	111.4 (3)
O3—N3—O4A	106.0 (7)	C6—C5—H5B	109.3
O3—N3—C17	120.2 (3)	C6—C5—H5A	109.3
O4—N3—O4A	33.1 (5)	H6A—C6—H6B	108.1
O4—N3—C17	108.2 (6)	C7—C10—C9	111.7 (2)
O4A—N3—C17	132.2 (7)	C7—C10—H10	124.1
N1—S1—C7	107.69 (13)	C8—S2—C7	90.39 (14)
N1—C1—H1A	108.0	C8—C9—C10	112.0 (3)
N1—C1—C2	111.5 (2)	C8—C9—C11	124.0 (3)
N1—C1—C6	109.9 (2)	C9—C8—S2	113.2 (2)
N2—C11—C9	113.8 (2)	C9—C8—H8	123.4
N2—C11—H11B	108.8	C9—C10—H10	124.1
N2—C11—H11A	108.8	C9—C11—H11B	108.8
N2—C12—C13	120.4 (3)	C9—C11—H11A	108.8
N2—C12—C17	123.6 (3)	C10—C7—S1	127.0 (2)
C1—N1—S1	120.60 (19)	C10—C7—S2	112.8 (2)
C1—N1—H1	119.7	C10—C9—C11	123.9 (2)
C1—C2—H2B	109.6	C11—N2—H2	117 (2)
C1—C2—H2A	109.6	H11B—C11—H11A	107.7
C1—C2—C3	110.3 (3)	C12—N2—H2	118 (2)
C1—C6—C5	110.4 (3)	C12—N2—C11	123.7 (3)
C1—C6—H6A	109.6	C12—C13—H13	119.0
C1—C6—H6B	109.6	C12—C17—N3	121.5 (3)
C2—C1—H1A	108.0	C13—C12—C17	115.9 (3)
C2—C3—H3A	109.4	C13—C14—H14	119.7
C2—C3—H3B	109.4	C13—C14—C15	120.5 (5)
H2B—C2—H2A	108.1	C14—C13—C12	121.9 (4)
C3—C2—H2B	109.6	C14—C13—H13	119.0
C3—C2—H2A	109.6	C14—C15—H15	120.0
C3—C4—H4B	109.3	C15—C14—H14	119.7
C3—C4—H4A	109.3	C15—C16—H16	119.8
C3—C4—C5	111.6 (3)	C15—C16—C17	120.4 (4)
H3A—C3—H3B	108.0	C16—C15—C14	120.0 (4)
C4—C3—C2	111.3 (3)	C16—C15—H15	120.0
C4—C3—H3A	109.4	C16—C17—N3	117.3 (4)
C4—C3—H3B	109.4	C16—C17—C12	121.1 (4)
C4—C5—H5B	109.3	C17—C16—H16	119.8
C4—C5—H5A	109.3		
			
S1—N1—C1—C2	−98.5 (3)	C1—C2—C3—C4	55.9 (4)
S1—N1—C1—C6	137.5 (2)	C2—C1—C6—C5	56.5 (3)
S1—C7—C10—C9	170.7 (2)	C2—C3—C4—C5	−55.4 (4)
S2—C7—C10—C9	0.7 (3)	C3—C4—C5—C6	55.0 (4)
S2—C8—C9—C10	0.6 (3)	C4—C5—C6—C1	−55.3 (4)
S2—C8—C9—C11	−176.0 (2)	C6—C1—C2—C3	−56.9 (3)
O1—S1—N1—C1	−56.2 (2)	C7—S1—N1—C1	57.9 (2)
O1—S1—C7—S2	−169.60 (16)	C7—S2—C8—C9	−0.2 (2)
O1—S1—C7—C10	21.0 (3)	C8—S2—C7—S1	−171.17 (18)
O2—S1—N1—C1	173.7 (2)	C8—S2—C7—C10	−0.3 (2)
O2—S1—C7—S2	−39.8 (2)	C8—C9—C10—C7	−0.9 (3)
O2—S1—C7—C10	150.8 (3)	C8—C9—C11—N2	−18.9 (4)
O3—N3—C17—C12	−3.6 (5)	C10—C9—C11—N2	165.0 (3)
O3—N3—C17—C16	177.7 (3)	C11—N2—C12—C13	−4.6 (5)
O4—N3—C17—C12	−173.6 (5)	C11—N2—C12—C17	177.0 (3)
O4—N3—C17—C16	7.6 (6)	C11—C9—C10—C7	175.7 (3)
O4A—N3—C17—C12	159.5 (9)	C12—N2—C11—C9	−75.0 (4)
O4A—N3—C17—C16	−19.2 (10)	C12—C13—C14—C15	−0.8 (6)
N1—S1—C7—S2	75.17 (19)	C13—C12—C17—N3	177.9 (3)
N1—S1—C7—C10	−94.3 (3)	C13—C12—C17—C16	−3.4 (5)
N1—C1—C2—C3	−180.0 (2)	C13—C14—C15—C16	−0.9 (6)
N1—C1—C6—C5	−179.5 (3)	C14—C15—C16—C17	0.3 (6)
N2—C12—C13—C14	−175.7 (3)	C15—C16—C17—N3	−179.3 (3)
N2—C12—C17—N3	−3.6 (5)	C15—C16—C17—C12	1.9 (5)
N2—C12—C17—C16	175.1 (3)	C17—C12—C13—C14	2.9 (5)

### Hydrogen-bond geometry (Å, °)

**Table:**

*D*—H···*A*	*D*—H	H···*A*	*D*···*A*	*D*—H···*A*
N1—H1···O1^i^	0.88	2.27	2.956 (3)	134.
N2—H2···O2^ii^	0.81 (3)	2.36 (3)	2.979 (3)	135 (3)
N2—H2···O3	0.81 (3)	2.01 (3)	2.620 (4)	132 (3)

Symmetry codes: (i) *x*, −*y*+1/2, *z*−1/2; (ii) *x*+1, −*y*+1/2, *z*+1/2.

## References

[bb1] Agilent (2011). *CrysAlis PRO.* Agilent Technologies, Oxford, England.

[bb2] Cuberes Altisen, R., Frigola Constansa, J. & Gutierrez Silva, B. (2007). US Patent Appl. US 20070066587 A1 20070322.

[bb3] Dolomanov, O. V., Bourhis, L. J., Gildea, R. J., Howard, J. A. K. & Puschmann, H. (2009). *J. Appl. Cryst.* **42**, 339–341.

[bb4] Santhakumar, V. & Tomaszewski, M. (2006). PCT Int. Appl. WO 2006052189 A1 20060518.

[bb5] Sheldrick, G. M. (2008). *Acta Cryst.* A**64**, 112–122. 10.1107/S0108767307043930 18156677

